# The Impact of COVID-19 and the Challenges of Post-COVID Rehabilitation in a Developing Country

**DOI:** 10.3389/fresc.2021.746061

**Published:** 2022-01-20

**Authors:** Taslim Uddin, Hasna Raihan Rahim, Md Nuruzzaman Khandaker

**Affiliations:** ^1^Department of Physical Medicine and Rehabilitation, Bangabandhu Sheikh Mujib Medical University, Dhaka, Bangladesh; ^2^Department of Physical Medicine and Rehabilitation, Combined Military Hospital, Dhaka, Bangladesh

**Keywords:** COVID-19 survivors, developing country, Bangladesh, people with disability, health sector impact, physical medicine and rehabilitation (PM&R), rehabilitation therapy, rehabilitation impact

## Abstract

The coronavirus disease 2019 (COVID-19) and its impact on human functioning are gaining increased interest. Like many other lower-income countries, the Bangladesh health and rehabilitation sector was adversely affected by COVID-19. Multiple challenges were identified for preparedness and medical rehabilitation during COVID-19 surges. Appropriate supervision of multispecialty long COVID clinics and attention to rehabilitation teamwork are important. Rehabilitation plays a key role in the management of patients with COVID-19 and can reduce the length of hospital stay and improve health outcomes. While waiting for people to be fully vaccinated; ensuring equitable access to COVID-19 vaccination, health care, and rehabilitation services among people with disabilities should be a part of the core mission during the pandemic. All levels of care including, critical, post-acute, or long covid clinic scale-up of rehabilitation services are needed. A physiatrist-led rehabilitation team approach is vital for the adaptation of rehabilitation interventions to improve the functional outcomes of persons with impairment and disability affected by COVID-19.

## Introduction

Knowledge continues to accumulate regarding the coronavirus disease 2019 (COVID-19), and its impact on human functioning is gaining increased interest. At this point of the pandemic, the appropriate supervision of multispecialty long COVID clinics and attention to rehabilitation during COVID-19 surges are important (1). Functioning is considered the third health indicator and the key indicator for rehabilitation (2). Earlier publications suggest that functional impacts of the COVID-19 pandemic are likely to involve far beyond the physical and mental health of the infected patient. Hence, the COVID-19 health systems need to be prepared accordingly to prevent and manage the functional impacts of COVID-19 at all levels of the health care system (3). According to the first global estimate of the need for rehabilitation services, about 2.41 billion people, in other words, 1 in every 3 people in the world needs rehabilitation at some point in the course of their illness or injury (3). Data from lower-income countries are scant. Indications suggest that rehabilitation is under-resourced, has not been prioritized in many countries, and long COVID clinics are often unavailable (1). Over 1,600 million adults in the world have a condition that would benefit from rehabilitation services, among them, about 77 million people live in the Southeast Asia region.

Non-communicable diseases (NCDs) lead to 15 million deaths annually worldwide, more than 85% of which occur in low- and middle-income countries (4). The existing burden of NCDs is further intensified with COVID-19 surges, the burden is profound in developing countries; which may differ between countries. There is emerging evidence that COVID-19 and long COVID disease frequently present with problems in mobility, sensory, cognition, or cardio-respiratory functions, many of these patients may need multiple medical specialties including rehabilitation at short or longer terms (1, 5). Rehabilitation practice is a combination of physical and rehabilitation medicine, occupational therapy, physiotherapy, speech and language therapy, orthotics and prosthetics, psychology, and evaluators of functioning interventions, including assistive technologies. Earlier studies demonstrated that rehabilitation in Bangladesh was struggling with the lack of a trained rehabilitation workforce and the serious crisis of space for expansion of services. The aim of this report is to present the impact of COVID-19 and its surges on the health and rehabilitation system in Bangladesh and to narrate the importance of rehabilitation teamwork to prevent and manage functional impairments of long COVID disease.

## Discussion

From March 2020 to July 2021, a period of 17 months has passed since the first case of COVID-19 was detected in Bangladesh. As of July 31, 2021, there were 1,249,484 confirmed cases and 20,685 reported deaths (6). The pandemic is currently categorized as being spread by community transmission, with more than 12,000 cases detected daily (7,450 cases/million population) with a detection rate of approximately 32% (6).

Bangladesh is a lower-middle-income country (LMIC) with a population of approximately 166 million. Its health system is poor and its annual health budget is <1% of the gross domestic product (GDP). There are 8 hospital beds, 5.6 doctors, and 1.07 nurses per 10,000 population. Bangladesh lags behind its neighbors in the health sector (7). About two-thirds of the total health expenditure is out-of-pocket, 65% of which is spent on medications. According to the WHO, the doctor-patient ratio in Bangladesh ranks second from last among South Asian countries. There remains a 36% vacancy in sanctioned health worker positions and only 32% of facilities have 75% or more of the sanctioned staff working in the facilities (8).

This shortage and the urban-oriented distribution of healthcare workers have made COVID-19 preparedness and mitigation difficult (9). Rehabilitation services for people with musculoskeletal conditions, stroke, spinal cord injury, and brain injury remain challenging. The registry of long-term disabling conditions including stroke, spinal cord injury, chronic pulmonary obstructive disease, or people with brain injury are not maintained (10) and rehabilitation teamwork is sidelined during COVID-19 surges. Most rehabilitation centers nationwide lack rehabilitation professionals, including occupational therapists, speech-language therapists, rehabilitation nurses, orthoptists, and other essential rehabilitation team members. Bangladesh physiatrist's accordance with the COVID-19 national directives stopped routine rehabilitation indoor admissions and therapy services. They joined the central pool of the COVID-19 roster for triage and, in association with rehabilitation therapists, they worked to maintain hybrid online-offline rehabilitation (5).

The attitude of most of the people of Bangladesh was strong; they believed that COVID-19 was controllable and a containable infection despite repeated surges (11). However, there was increased anxiety and fear among frontline service providers owing to the high death rate among doctors and its impact on the provision of quality care. Approximately 3,000 doctors and 2,500 nurses in Bangladesh have been affected by the coronavirus, of whom approximately 150 doctors have died of COVID-19, including an Associate Professor of Physical Medicine & Rehabilitation (PM&R) at the Medical University (12). Only 18.3% of doctors, nurses, and midwives have been trained on infection prevention and control (IPC) practices (13). The high rate of infection could be due to the poor rate of IPC training, enormous workload, and poor quality of supplied personal protective equipment.

Rehabilitation care includes a comprehensive range of inpatient and outpatient services and complex continuing care for adults who experience debilitating illnesses or injuries. However, rehabilitation was not a health priority in LMICs. Manpower, fund placement, the space crisis for expansion, and adaptation of rehabilitation services were the major challenges to continue rehabilitation consultation and therapy services (14). Bangladesh is also currently additionally burdened by the largest refugee camps in the world. These people are also affected by COVID-19 and many may require PM&R services. People with COVID-19 face a crisis in the availability of hospital beds and availability of oxygen therapy equipment, including prescribed medications. Intensive care units are deficient in human resources and most are run by uni-specialty teams, although COVID-19 is a multisystem disease. Post-COVID follow-up clinics initially appeared to be promising for both academic purposes and patient services; however, their attraction gradually decreased owing to deficient manpower and increased workloads (15). Other issues included poor awareness regarding timely referral to rehabilitation services and poor interprofessional relationships concerning rehabilitation team function among neurologists, rheumatologists or orthopedic surgeons, and rehabilitation physicians.

People who have suffered the effects of this disease might be at increased risk of long-term impairment and disability. Post-acute rehabilitation and rehabilitation needs are also specific to individualized conditions of immobility and physical and cardio-respiratory deconditioning, including those with long COVID syndrome. Rehabilitation interventions address the consequences of COVID-19 survivors, including cognitive, physical, and psychiatric impairments. Short and individualized therapeutic exercise programs were shown to improve functional status (16). However, access to medical and rehabilitation services by people with disabilities was not prioritized during the lockdown restrictions and there was no special arrangement for this population in the COVID-19 vaccination campaign. There are concerns regarding increased risks of coronavirus infection and the likelihood of poor health in persons with disabilities.

People with activity limitations and disabilities had greater challenges in maintaining their medication supplies and continuing physical and occupational therapy. Home care is especially important in situations where telemedicine and telehealth technologies are especially effective, such as during epidemic surges and lockdown restrictions. A large developing country reported that more than 50% of the rehabilitation centers provided telerehabilitation for neurological disorders during the COVID-19 pandemic (17). The telerehabilitation for neurological disorders included patient screening and monitoring of disease status to detect the early signs of deterioration, provide prompt treatment, provide rehabilitation medicine consultation, and plan therapy modalities, with real-time assessment of clinical status (18).

Ensuring equitable access to safe and adequate COVID-19 vaccinations, health care, and rehabilitation services among people with disabilities should be a part of the core mission during the pandemic. The government of Bangladesh has worked to increase capacity building to ensure a safe and effective roll-out of the COVID-19 vaccine nationwide; however, as of July 19, 2021, only approximately 2.7% of the population were vaccinated (19). High prevalence rates of vaccine refusal and hesitancy were observed in slum and rural populations, with higher rates in elderly people and people with NCDs; thus, these populations require urgent attention to develop effective vaccine campaigns (11). While waiting for full vaccine coverage or expected herd immunity leading to near normal life, hybrid on and offline consultation and continuation of rehabilitation therapy services are recommended to maintain gait training, spasticity-specific therapy, and preventive measures for pressure injury.

## Conclusions

The coronavirus disease 2019 (COVID-19) and long COVID disease added further workload to the existing burden of disability in Bangladesh. Despite multiple constraints, the attitude of the general public of Bangladesh remained positive. Online and offline rehabilitation care adapting the central COVID-19 duty rosters were practiced. To address the multi-organ-involved long COVID disease, well-organized and coordinated multi-specialty teams are required. For the functional achievement of COVID-related impairments, early involvement of physiatrist-led multidisciplinary rehabilitation teams with scale-up of rehabilitation services is recommended.

## Data Availability Statement

The original contributions presented in the study are included in the article/supplementary material, further inquiries can be directed to the corresponding author/s.

## Author Contributions

TU conceived the research theme, organized the initial planning in discussions with the co-authors, and finalized the manuscript. HR searched for relevant information and contributed to the manuscript development and organization. MK coordinated the information search, contributed to the development of the manuscript, and reviewed the manuscript. All the authors finally approved the manuscript for submission.

## Conflict of Interest

The authors declare that the research was conducted in the absence of any commercial or financial relationships that could be construed as a potential conflict of interest.

## Publisher's Note

All claims expressed in this article are solely those of the authors and do not necessarily represent those of their affiliated organizations, or those of the publisher, the editors and the reviewers. Any product that may be evaluated in this article, or claim that may be made by its manufacturer, is not guaranteed or endorsed by the publisher.
